# Parvoviruses NS1 oncolytic attributes: mechanistic insights and synergistic anti-tumor therapeutic strategies

**DOI:** 10.3389/fmicb.2025.1631433

**Published:** 2025-08-11

**Authors:** Abdul Haseeb, Wafa Yousaf, Zhigang Cao, Kuohai Fan, Na Sun, Panpan Sun, Yaogui Sun, Huizhen Yang, Wei Yin, Hua Zhang, Zhenbiao Zhang, Jia Zhong, Jianzhong Wang, Hongquan Li

**Affiliations:** ^1^Shanxi Key Laboratory for Modernization of TCVM, College of Veterinary Medicine, Shanxi Agricultural University, Taigu, China; ^2^Laboratory Animal Center, Shanxi Agricultural University, Taigu, China

**Keywords:** parvoviruses, NS1, cell cycle arrest, apoptosis, oncolysis, immunomodulation, genetic engineering, combinational therapy

## Abstract

Parvovirus is comprised of a single-stranded DNA structure, encompassing distinct structural and non-structural proteins. Structural proteins are referred as viral proteins, which facilitate for the viral capsid. Among non-structural proteins, NS1 is the most significant, exhibiting substantial characteristics related to viral replication, pathogenicity, and is notably recognized for its remarkable oncolytic properties. NS1 possesses a distinctive structure; however, it differs across different parvovirus species. It is comprised of three fundamental domains: the N-terminal origin binding, helicase domain, and C-terminal domain, all crucial for significant functions. In several parvovirus species, such as CPV, MVM, BPV, and HPV-B19, NS1 halts the cell cycle at distinct stages, including G1, G2, and S phases of the life cycle, and induces cell death. Predominantly, parvovirus NS1 has also been significantly recognized to induce tumor cell death *in vitro* and *in vivo* by following different mechanisms, including cytotoxicity, autophagy, immunomodulation, mitochondrial depolarization, and most significantly, apoptosis. This may lead to several intracellular changes, including reactive oxygen species (ROS) level, mitochondria, PARP, caspase, and their subtype activation, ultimately leading to DNA and other cellular level changes, which facilitate apoptotic cell death. These characteristics of NS1 and its combinational therapy revealed a wide range of evidential research that demonstrated its anti-tumor effects through several pathways and can even induce a substantial activation of the immune response. This review mainly aims to elucidate the oncolytic attributes of parvoviral NS1, focusing on its capabilities and the mechanism demonstrated in prior research. It also addresses genetic engineering and combinational therapy aimed at augmenting the oncolytic efficacy of NS1 for more potent application as a tumor therapeutic agent. The increasing focus on virotherapy and precision oncology underscores the necessity for thorough exploration of the molecular mechanisms, delivery techniques, and clinical implications of NS1, thereby facilitating the development of innovative, tumor-selective anticancer approaches.

## 1 Introduction

The *Parvoviridae* family is generally categorized into three distinct subfamilies*: Densovirinae, Parvovirinae*, and *Hamaparvovirinae* (Pénzes et al., 2020). Members of the subfamily *Densovirinae* and *Parvovirinae* can infect both invertebrates and vertebrates; however, the newly established subfamily *Hamaparvovirinae* also has the capability to induce severe disease in both vertebrates and invertebrates (Afumba et al., 2022). Parvoviruses belonging to the subfamily *Parvovirinae* are structurally small, non-enveloped, icosahedral viruses characterized by a linear directional single-stranded DNA (ssDNA) genome comprising approximately 5,000 base pairs, with a diameter ranging from 18 to 26 nm (Gupta et al., 2016; Liu and Kirn, 2008; Xie et al., 2023; Saxena et al., 2013). Taxonomically, this subfamily encompasses three clinical and biological significant genera: (i) Bocaparvovirus featuring human bocavirus (HBoV) with a diameter of ~26 nm, icosahedral capsid with surface spikes, identified as emerging pathogens leading to severe respiratory (Wang et al., 2022) and gastrointestinal infections in humans and capable of modulating host cell cycle progression; (ii) Protoparvovirus having ~22 nm diameter, smooth capsid with conserved canyon structure, which includes highly virulent animal pathogens like canine parvovirus (CPV), feline panleukopenia virus (FPV) and mink enteritis virus (MEV), that have evolved through host-range mutations in their VP2 capsid protein and induce host cell tropism, causing devastating hemorrhagic enteritis in their respective hosts; (iii) Dependoparvovirus with ~25 nm diameter, capsid with 60 monomer units and having VP3-dominated structure (e.g., adeno-associated viruses (AAV), which necessitate helper viruses (e.g., adenoviruses) for replication and have been utilized as gene therapy vectors (Campbell et al., 2022) owing to their non-pathogenicity and tissue-specific serotypes; and (iv) Erythroparvovirus, predominantly human parvoviruses B19 having ~26 nm diameter with icosahydral symmetry. In the direction of proteomics study, the Parvoviral genome comprises of two open reading frames (ORFs) and overlapping genes consisting of a total of four proteins among which there are two Non-structural proteins (NS1 and NS2) along with two major contributing structural proteins or viral proteins (VP1 and VP2; Decaro and Buonavoglia, 2012), later studies also revealed about VP3 viral protein in canine species (Li et al., 2017). These viral proteins contribute to the formation of the capsid structure of viruses and organize it into an icosahedral shape. It creates specific loops and folds, resulting in a hairpin-like structure responsible for its replication (Cotmore et al., 2019), which can interact with the heparin sulfate of the host. The non-structural proteins exhibit significant homologous sequence arrangements and are considered the main causative agent of parvoviral cytotoxicity in cells, with NS1 having a major impact (Iseki et al., 2005). The viral NS1, comprising 672 amino acids, serves a fundamental function in regulating the parvoviral life cycle. It is directly engaged in several key functions, including the binding and hydrolysis of adenosine triphosphate, site-specific DNA binding, DNA cleavage, helicase activity, and transregulating promoters (Marchini et al., 2015; Wang et al., 2012). These features enable NS1 to regulate various processes essential for progeny particle generation, encompassing viral DNA amplification and gene expression (Wang et al., 2012).

In several *in vitro* and *in vivo* investigations, numerous parvovirus genus species have been recognized as having oncolytic attributes, particularly the *rat parvovirus* H-1 (H-1PV; Angelova et al., 2024), *minute virus of mice* (MVMp), *Human parvovirus* (B19V) have attained significant attention for their possible applications as anti-tumor agents and can penetrate deep into the cancer cells of the infected tissue part resulting into efficient destruction of cancerous cells along with suppression of tumor cells growth in in-vivo animal models (Bretscher and Marchini, 2019; Arora et al., 2021). H-1PV is a single-stranded, non-enveloped DNA virus originating from rats, known for its ability to selectively target and eliminate a wide variety of human cancer cells from diverse origins (Manocha et al., 2021), as well as exerting oncosuppressive effects on several other cancer cells *in vitro* (Ferreira et al., 2020). Because of fundamental oncotropism and oncosuppressive properties, a range of strategies has been employed to enhance the anticancer potential of oncolytic parvoviruses, such as the creation of second-generation parvoviruses that exhibit improved oncolytic and immunostimulatory effects, as well as the strategic combination of parvoviruses with other therapeutic approaches (Hartley et al., 2020). Meanwhile, there are also some other viruses exhibiting considerable oncolytic potential that have been recognized as promising options for clinical use. *Chicken anemia virus* (CAV) and *Canine parvovirus* (CPV) exhibit inherent, intrinsic oncolytic characteristics (Bär et al., 2013; Kalafati et al., 2023). It has been noted that a variety of oncolytic viruses and their associated viral proteins exhibit a remarkable ability to eliminate tumor cells with minimal or negligible adverse effects on normal cells (Gupta et al., 2016; Lu et al., 2024). This specified characteristic in oncolytic viruses can be attained with the aid of genetic engineering by the deletion of viral genes essential for replication in normal cells or by incorporating the tumor tissue-specific replication promoter or genes, enabling replication exclusively in tumor cells (You et al., 2004). Parvoviruses enhance the lysosome membrane permeability, allowing lysosomal enzymes such as cathepsin to infiltrate the cytoplasm and induce death in tumor cells by activating cell death-inducing factors (Reddy et al., 2023). Notably, certain mechanisms, such as apoptosis in CPV2 are followed to promote cell death (Neyestani et al., 2025). Parvoviruses have been recognized in several studies as possessing tremendous oncolytic properties attributed to their oncotoxic proteins. Specified proteins such as the non-structural protein 1 (NS1), a constituent of the rat parvovirus H1 (H1-PV), offer a unique approach to the therapy of malignancies that are unresponsive to previous treatments (Hauswirth et al., 2023). NS1 of *human parvovirus* typically induces caspase-dependent apoptosis of the cancerous cells, leading to cell cycle arrest in the G1 phase. In MVM, NS1 induced cell cycle arrest due to the formation of nicks in the chromatin part of cells and also induced structural alterations in the cytoskeleton (Nüesch and Rommelaere, 2007). Regarding the canine family, *Canine parvovirus* also possesses this distinctive NS1 protein, which exhibits an effective therapeutic effect on the tumor cell lines (Xie et al., 2023). Mechanistically, parvoviral NS1 exhibits considerable oncolytic efficiency by multifunctionally inducing DNA damage, cell cycle arrest, and apoptosis in neoplastic cells while sparing normal tissues. NS1, functioning as a site-specific endonuclease, preferentially cleaves and destabilizes the genomic DNA of rapidly growing cancer cells, therefore exacerbating replication stress and activating the DNA damage response (DDR) pathway. Additionally, NS1 disrupts essential oncogenic signaling pathways, such as the PI3K/Akt and NF-κB pathways, while promoting immunogenic cell death (ICD) via the generation of damage-associated molecular patterns (DAMPs) and tumor-associated antigens, thereby enhancing antitumor immunity. The ability to impede homologous recombination (HR) repair increases the vulnerability of cancer cells to NS1-induced cytotoxicity, making it a potential option for targeted oncolytic virotherapy, particularly in tumors with deficient DNA damage response (DDR) pathways.

NS1 is a multifunctional nuclear phosphoprotein that plays an essential role in the virus replication cycle. It is an essential component in viral DNA replication and possesses distinct roles in the viral life cycle (Niskanen et al., 2013). Along with it, expression of the NS1 protein of different parvoviruses has also been observed in resulting cell death through apoptosis induction (Saxena et al., 2013), as well as through other mechanisms such as autophagy, immunomodulation, and cellular toxicity. Intracellular NS1 protein accumulation is one of the significant factors in the cytotoxicity inflicted by viruses in tumor cells, which is attributed to its bipartite nuclear localization sequence (NLS) that extends from amino acid residues 194 to 216. NLS serves as a signal source for nuclear localization that ultimately facilitates the nuclear entrance of the NS1 protein which is essential for the oncolytic effects (Marchini et al., 2015). Moreover, the alteration of certain residues (Thr-435 and Ser-473) in NS1 is also crucial for the cytotoxicity against cancer cells leading to cell death, which was partially mediated by the dysregulation of intracellular signaling pathways (Wyatt et al., 2019).

Oncolytic viruses can initiate various forms of cell death, such as apoptosis, oncosis, and necrosis, leading to tissue damage and facilitating the progression of the infection (Zhou et al., 2017; Imre, 2020). Apoptosis represents the predominant and thoroughly examined type of cell death triggered by viruses, which is a programmed death of cells in multicellular living organisms, typically during the process of aging. It is also a part of the defense mechanism that initiates in response to extrinsic damaging or harmful factors with viral proteins being particularly significant among various stimulating agents (Saxena et al., 2013). It encompasses the activation of different ion channels, as well as the expression and regulation of different genes. Generally apoptosis mechanism involves two prominent pathways: the extrinsic pathway and the intrinsic pathway. Both pathways are stimulated by the same effector caspases that intensify the initial cell death and initiate death signals. There are specific tumor necrosis factor receptors (TNFR) that can play a role in the activation of caspase 8 and caspase 10, which as a result, induce apoptosis (D’arcy, 2019). For the induction of apoptosis cell death, specific ligands bind to these TNFR receptors and induce the recruitment of Fas and TNFR-associated death domain proteins, ultimately leading to the formation of a death-inducing complex (DISC) which results in the activation of caspase 8 (Nüesch et al., 2008). *Sol et al.* proposed in a study that a potential correlation also exists between the *human parvovirus* B19 NS1 protein and apoptotic pathways induced due to TNFr-α in human erythroid cells (Sol et al., 1999). TNF-α signaling induced by *human B19 parvovirus* NS1 protein can also trigger the activation of IKK-α, IkB, and NF-kB (Tsai et al., 2013). It was observed that apoptotic cell death induced by NS1 protein was dependent on caspase and independent of p53 (Hristov et al., 2010). Until now, numerous investigations have elucidated the mechanism of intrinsic apoptotic tumor cell death characterized by mitochondrial depolarization, the release of cytochrome c, and activation of the caspase, particularly caspase 9 induced by NS1 protein of variouse strains of parvoviruses.

Tumor-eliminating viruses preferentially reproduce within cancer cells, resulting in their destruction upon completion of the replication cycle. Three different categories of oncolytic viruses have been employed till now in clinical trials: (i) Oncolytic viruses possessing inherent anti-neoplastic characteristics, (ii) oncolytic viruses altered to stimulate the immune response as immunotherapeutics (Seyed-Khorrami et al., 2023), and (iii) oncolytic viruses engineered to tumor-selective reproduction (Carter et al., 2021). Live viruses were initially employed in cancer treatment over a hundred years ago. However, promptly after its inception, the approach had to be discontinued due to concerns over toxicity, emergence of antiviral immunity, and potential comeback of hazardous viruses (Liu and Kirn, 2008). Recently, there has been an upsurge in focus on the development of virus-based cancer therapies within the scientific community. Despite the progression of oncolytic viruses in clinical trials, their efficacy as nanotherapeutic agents has not been as expected. Recent experimental evidence suggested that genetically altering these viruses can enhance their oncolytic capabilities (Cristi et al., 2022). The advancements in molecular biotechnology have enabled the genetic alterations of viruses to enhance their selectivity for tumor cells, a technique that was primarily illustrated through the use of Herpes Simplex virus type-1 (HSV-1) in an experimental glioma model (Jamal and Oglu, 2018).

## 2 Parvovirus structural illustration

**Figure 1:**
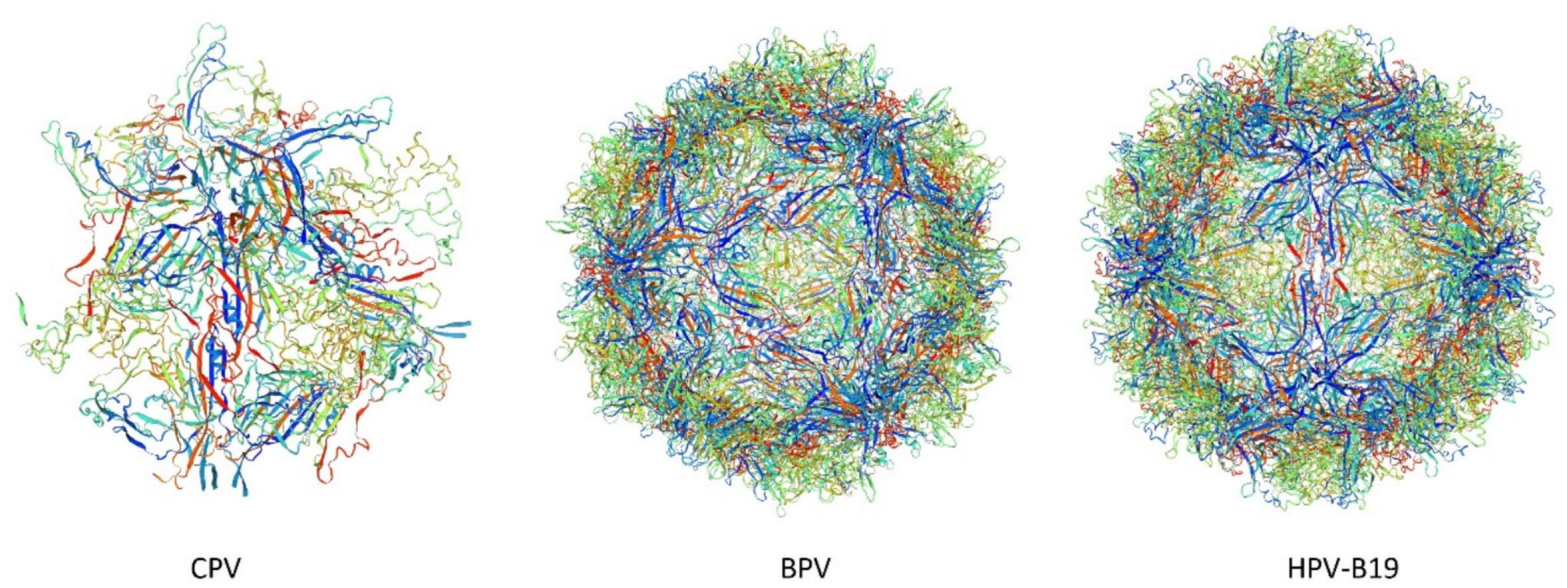
3D structures of *Canine Parvovirus* (CPV), *Bovine Parvovirus* (BPV), and *Human Parvovirus* B19 (HPV-B19) created by Swiss-Model (accession no B2ZG07 | SWISS-MODEL Repository, P07297 | SWISS-MODEL Repository, P07299 | SWISS-MODEL Repository).

The structures of parvoviruses exhibited conserved features while also displaying unique characteristics that contribute to viral density replication. The capsid typically possesses standard structural features, such as surface depressions at the two-fold axis, protrusions at the three-fold axes, and channels at the five-fold axis (Jabbari et al., 2025). As illustrated in Figure 1, the folding structures of *Canine Parvovirus* (CPV), *Bovine parvovirus* (BPV), and *Human parvovirus* (HPV-B19) are nearly identical, although they exhibit variations in the local surface-specific domains like the Icosahedral five-fold axis, icosahedral three-fold axis, and icosahedral two-fold axis, respectively, which makes them distinctly distinguishable from one another. The CPV2 genome is comprised of two coding frames; the 3′ end encodes the non-structural proteins NS1 and NS2, while the 5′ end encodes viral capsid proteins VP1 and VP2 (Hao et al., 2022). However, subsequent studies revealed that mature viruses possess an additional viral protein VP3 (Dik et al., 2022), which is generated as a result of the hydrolysis of the VP2 capsid protein. Research on the NS1 protein of parvoviruses, specifically CPV has remained limited throughout the virus’s evolutionary history. It is a characteristically phosphorylated protein possessing tumor therapeutic properties following apoptosis and oncolysis (Miao et al., 2022). The comparative similarity index of *Canine parvovirus* NS1 amino acid sequence with mouse and rat parvovirus NS1 was approximately 60%, whereas compared to *human parvovirus* was less than 20% (Wang et al., 2020). CPV is the most prevalent viral infection of the family exhibiting pathogenic traits to affect multiple organs of the same infected animal and having the most prominent symptom of gastroenteritis leading to severe illness (Kilian et al., 2018) Currently, four different antigenic types are circulating throughout the world that are CPV2, CPV2a, CPV2b, and CPV2c which are emerged due to mutation (Dik et al., 2024). In this viral infection, symptomatic conditions like gastroenteritis arise from mutation of about a minimum of 6–7 amino acid residues, which are M87L, I1o1T, S297A, A300G, D305Y, N426D, N426E, and V555I in the VP2 protein. This VP2 protein is specifically involved in interaction with the Transferrin receptor (TfR) of the host cell (Miranda and Thompson, 2016; Vannamahaxay et al., 2017). Particularly, these Transferrin receptors (TFR) contributed to the evolution of the CPV-2 strain, which likely emerged through mutations-enabled interaction with this receptor (Miranda and Thompson, 2016). As far as the mutation firms, the viral affinity characteristic of the canine TFR receptor was enhanced and mediated efficient infection to a significant degree (Kaelber et al., 2012).

The analysis of the VP2 gene revealed that it has a 1755 bp length encoding 584 AA and the capsid of the virus comprised nearly 60 structural proteins with the VP2 protein being the most significant, constituting 90 percent of the capsid of the virus and influencing the host range, pathogenicity, and antigenic characteristics of the virus (Shackelton et al., 2005). This VP2 gene also significantly contributes to the mutation and evolution of different antigenic types of CPV (Temizkan and Sevinc, 2023). The antigenic drift in the VP2 gene may also lead to severe resistance problems like Vaccine failure, which can potentially elevate its incidence across conducive environments (Llamas-Saiz et al., 1996). It has been reported that three mutational sites of the VP2 genome mainly lead to the condition of vaccine failure; these sites are F267Y, Y324I, and T440A (Allison et al., 2014). As shown in Figure 1, these VP2 proteins aggregate and contribute to the icosahedral structure formation of parvovirus species. The 3D model structure of CPV, BPV, and HPV-B19 has notable variations in the compactness and symmetry as evidenced by their color-coded proteins and secondary structure elements, including β-strands, α-helices, and loop configurations. CPV exhibits a relatively loose and less compact structure characterized by an indistinct five-fold axis, indicating reduced stability. BPV exhibits a more structured icosahedral arrangement with well-defined five-fold and three-fold symmetry. HPV-B19 exhibits the highest structural complexity characterized by a densely interwoven capsid and distinct 5-fold, three fold and two fold axis which contributes to its enhanced stability and infectivity. The progression of the folding and symmetry from CPV to HPV-B19 illustrates the evolutionary adaptations of these viruses, influencing their capsid rigidity, host interactions and environmental persistance.

## 3 NS1 and its structure

**Figure 2:**
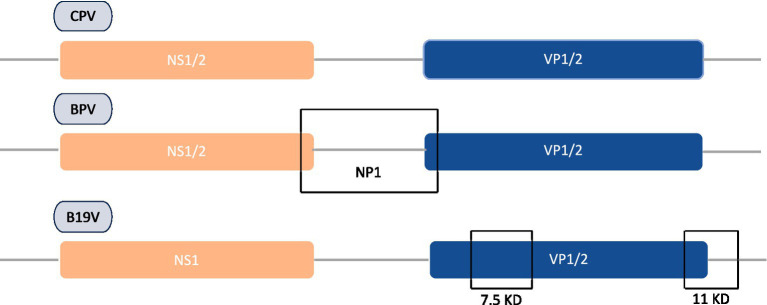
Comparative genomic organization of CPV, BPV, and HPV-B19 proteins encoding regions along with unique VP variations in B19 parvovirus.

**Figure 3:**
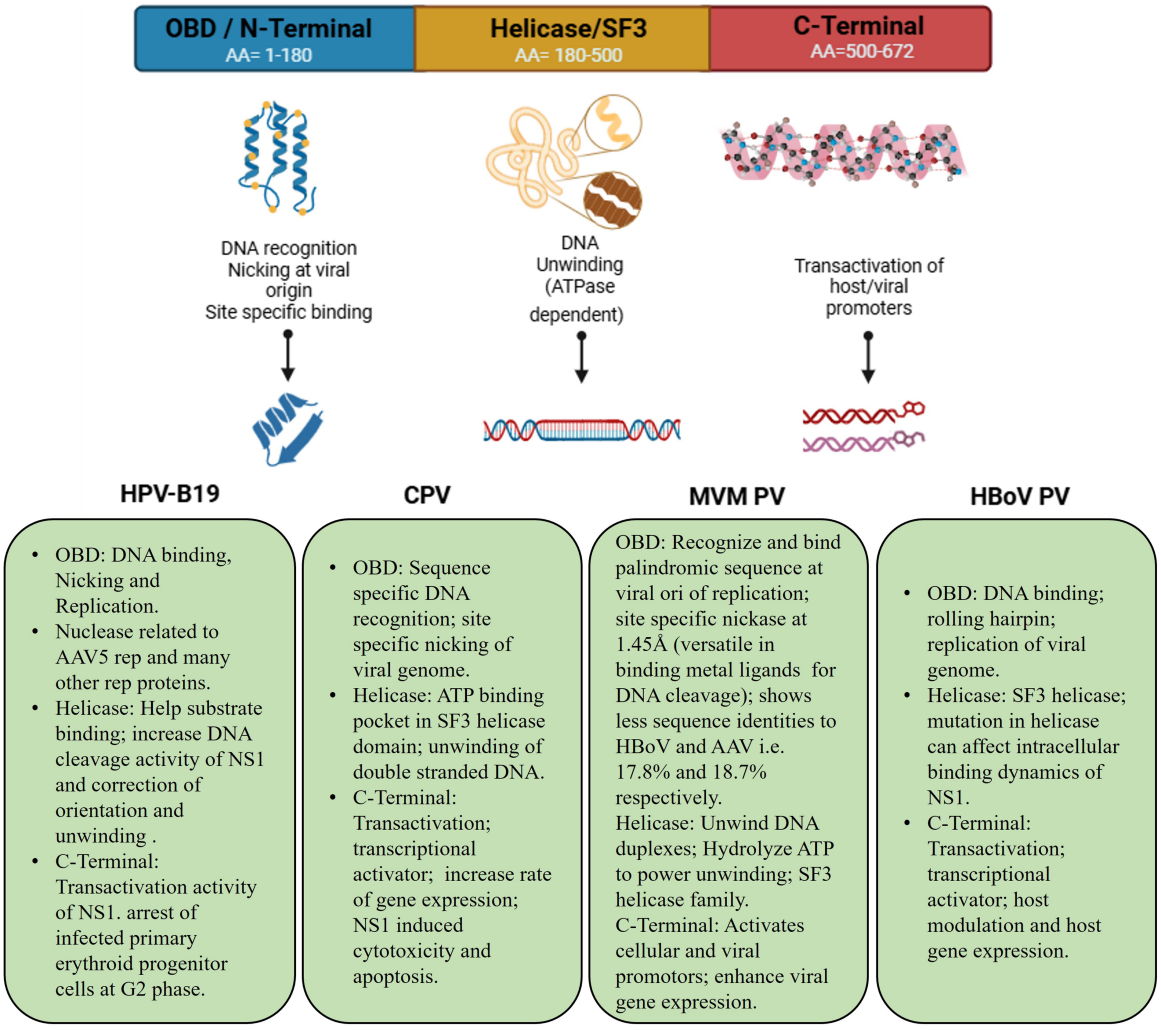
Schemetic bilogical striucture illustration of parvoviral NS1 and functional significance across species. Amino acid composition of each domain and its common significant roles in cellular biological processes.

Concerning genomic protein organization of parvoviral species, CPV and BPV are often comprised of analogous NS1/NS2 proteins, while HPV-B19 solely encodes NS1. Furthermore, BPV possesses an additional NP1 protein that is lacking in both CPV and HPV-B19. This NP1 protein plays a vital role in efficient viral gene expression by regulating mRNA processing and nuclear export. Figure 2 demonstrates that the VP1 and VP2 are conserved in all three viruses, with HPV-B19 exhibiting an additional weight distinction (7.5 kDa and 11 kDa) within the VP1/2 region, implying potential post-translational modifications or cleavages. These genomic variations indicate functional adaptations among parvovirus species affecting replication, host range, and pathogenicity. NS1 is a non-structural protein of parvovirus having a molecular weight of about 76.7 kDa nuclear phosphoprotein which plays a crucial function in the life cycle of the virus (Kwak et al., 2023). In recent years, there have been significant advancements in the binding configurations of the NS1 protein to DNA sequences, coupled with modifications in its molecular properties and viral gene expressions related to infections. In view of structural biology insights, parvoviral NS1 is comprised of three most significant domains: N-Terminal/OBD, Helicase, and C-Terminal/transcription activation domains. These domains have significant functional and structural roles in several parvoviral species like genome replication, DNA unwinding, transactivation, which has been elaborated in Figure 3. Structural analysis of the terminal regions indicates that the N-terminal of the NS1 proteins holds significant importance, and it encompasses a specific origin of replication binding (OBD) domain (Gorbalenya et al., 1990). This OBD enables NS1 to recognize and bind to the origin of replication within the viral genome. The N-terminal is also referred to as the DNA binding domain or site of nuclease, while the central region comprises of helicase site containing the NTP Binding site, and the terminus C contains the transactive domain that plays a key role in transactivation (Niskanen et al., 2013; Ganaie and Qiu, 2018; Niskanen et al., 2010). The N-terminal of NS1 comprises site-specific binding to dsDNA, having overlapping characteristics. It also has an NLS signal system, named a Nuclear localization signal, that guides its transportation to the nucleus of the host (Chen et al., 2024). Regarding DNA domains, there are typically two in number: The N domain and the Helicase domain, which play a crucial role in the replication of the viral genome and are also involved in the regulation of the production of essential proteins. Moreover, the Non-structural 1 protein also exhibits a key function in the replication cycle of the virus, pathogenic effect, and DNA Packing.

**Figure 4:**
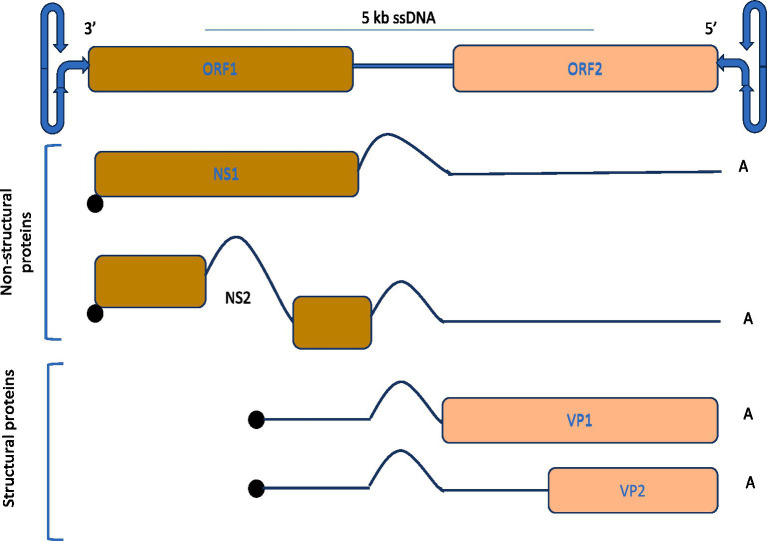
Genomic organization and protein expression strategy of parvoviruses, and alternative splicing for efficient replication.

The genomic layout and expression mechanism of parvovirus is illustrated in Figure 4, characterized by approximately 5 kb ssDNA and comprised of non-structural proteins encoding regions comprised of NS1 and NS2, which play vital roles in host interaction, and structural proteins encoding regions having VP1 and VP2, which constitute the viral capsid. The parvoviral genome is flanked at both the 3′ and 5′ ends by distinctive hairpin structures, collectively referred to as the “rolling hairpin” configuration, which are essential for genome replication and regulation. The mechanism of alternative splicing regulates the expression of these proteins as indicated by intronic regions and distinct polyadenylation sites. During this process, NS1 facilitates DNA replication within the genomes by binding to specific sites, most notably the origin on the right side of the viral structure, and culminates in strand and site-dependent nicking of viral DNA (Tewary et al., 2015).

After entering the host cell nucleus, genomic DNA transforms into a double-stranded replication form by synthesizing complementary DNA strands. The subsequent step involves the production of the NS1 protein and its transcription of the protein. This protein leads to viral DNA replication as well as regulates and coordinates host cellular activities to facilitate viral replication (Niskanen et al., 2010). Besides dsDNA, the core region of parvovirus NS1 also possesses the capability to fully function as an ssDNA helicase. Generally, Helicase is a type of enzyme that utilizes energy generated as a result of the hydrolytic process of ATP to displace or replace DNA or RNA and untwist the double-stranded region structures of nucleic acids (Cotmore and Tattersall, 2013). Helicases have been categorized into three main subfamilies (SF1, SF2, and SF3) depending on the sequence variation. Among these, the NS1 protein is associated with the SF3 subfamily, whose members are typically encoded by small DNA and RNA viruses (Singleton and Wigley, 2002; Zarate-Perez et al., 2013). In terms of structure, the SF3 has two domains, the first is the DNA origin interaction domain (OID) and the second is the AAA + motor domain, of which the AAA + motor domain is a structure-specific attribute of cellular initiation and initiator oligomerization (James et al., 2003). This SF3 subfamily is also comprised of 4 conserved limited sequence motifs, which are termed as Walker A, B, B′, and C. These walkers are confined within a limited region of 100 amino acids. Among these walkers, walker B′ is the amino acid region consisting of 14 amino acids, while Walker C is specifically for the residues (Iyer et al., 2004). All of these walkers are involved in the formation of the core of active sites of enzymes (Hickman and Dyda, 2005).

**Figure 5:**
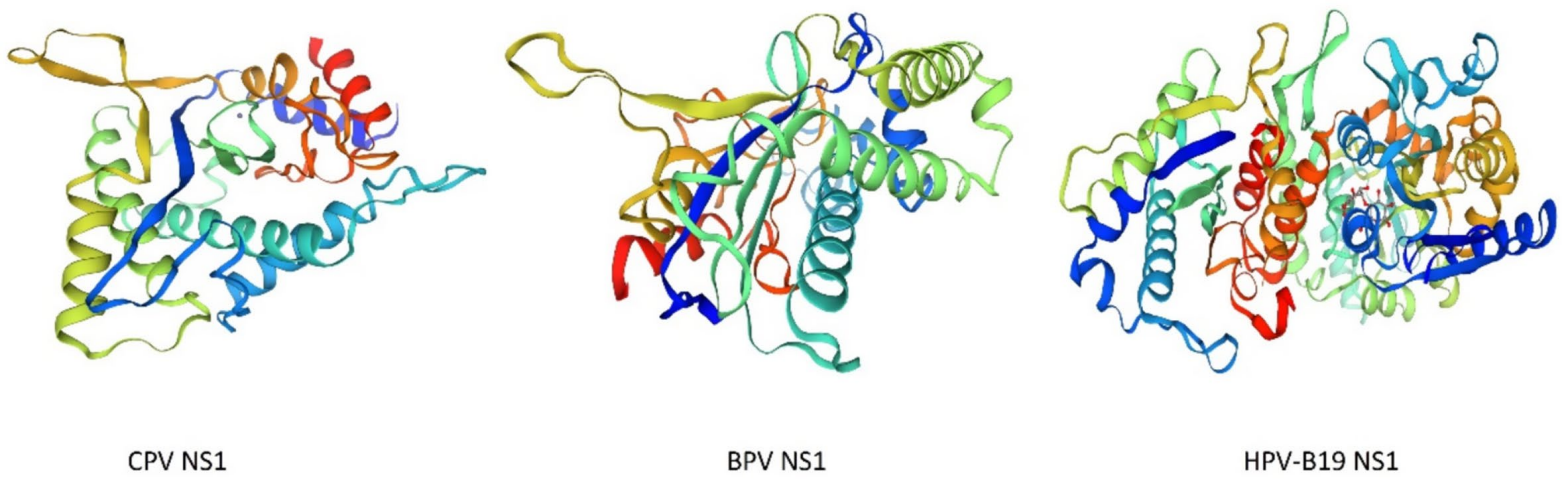
3D structures comparison of NS1 protein of CPV, BPV, and HPV-B19 created by Swiss model (P12929 | SWISS-MODEL Repository, P07296 | SWISS-MODEL Repository, 6usm.1 | SWISS-MODEL Template Library), revealing ATPase folds with host conformational adaptations and specificity.

The structural and functional unification in the *Parvoviridae* family can be recognized by the amino acid sequence arrangement of MEV, CPV, MVM, and PPV and their firm preservation of the residues located at the active sites (Mansilla-Soto et al., 2009). A 3D structural comparison of NS1 proteins of CPV, BPV, and HPV-B19, shown in Figure 5, demonstrates distinct conformational variations that likely influence their functional divergence and host specificity. Each structure possesses a conserved overall fold comprising α-helices and β-sheets, which are characteristics of the AAA + ATPase domain essential for viral replication. The CPV NS1 structure appears to be compact, featuring a well-ordered arrangement of essential domains, indicating a stable conformation optimized for DNA binding and ATPase activity in hosts. In contrast, the BPV NS1 displays more extended loops and relatively more flexible configurations, potentially enhancing interaction with the host factors. The HPV-B19 NS1 structure appears to be the most complex in comparison, displaying a most intricate arrangement of helices and loops. This arrangement potentially enables protein–protein interaction necessary for replication in host target cells in humans. These structural differences reflect evolutionary adaptations for pathogenicity and viral replication, reflecting the diverse mechanisms by which parvoviruses exploit the cellular environment. The compactness of the parvovirus B19 NS1 structure may correlate with its nuclear localization in host cells, while CPV and BPV may have different host adaptive strategies.

## 4 NS1-mediated cell cycle arrest

The cell cycle is a series of events that governs cell growth and division (Uzbekov and Prigent, 2022), comprising distinct phases named interphase, mitotic phase, and resting phase, each with a specific function and regulatory mechanism. The NS1 protein of various viruses serves an important function in influencing cell cycle arrest by playing a significant role in different stages of the cell cycle. Predominantly, NS1 is majorly localized in the nucleus of the virus-infected cells, but a small fraction can also be found in the cytoplasm, indicating the presence of one or two Nuclear localization sequences (NLS; Hale et al., 2008). As a result of its bipartite nuclear localization sequence (NLS) comprising amino acid residues 194 to 216, the NS1 protein accumulates inside cells, which significantly aids and enhances virus-induced cytotoxicity in neoplastic cell lines (Manocha et al., 2021). Furthermore, the cytotoxic effects of NS1 on cancer cells are facilitated by the dysregulation of intracellular signaling pathways (Malla et al., 2020), which occurs due to the modification of specific residues (Lysine 405 to Serine) in NS1 (Manocha et al., 2021; Malla et al., 2020; Li and Rhode 3rd, 1990). In addition to inducing cytotoxicity and cell death, Parvovirus NS1 also induces cell cycle arrest to facilitate viral genome replication (Chen and Qiu, 2010). To ensure the smooth progression of their replication cycle, viruses rely on continuously infecting the host cell and utilize the host cell’s replication machinery to duplicate the viral genome, ultimately achieving their reproductive and survival objectives. Numerous investigations have demonstrated that parvovirus NS1 may halt the cell cycle and facilitate the consistent replication of the viral genome. Simultaneously, it was revealed that parvovirus NS1 is associated with several inhibitor complexes and cell cycle regulators including cyclin-dependent kinase (CDK) inhibitor p21CIP1, Cyclin A, CDK, Cyclin E, and CDK2, which leads to cell cycle arrest (Chen et al., 2024; Op De Beeck et al., 2001). Along with it, certain cellular proteins can also influence the cell cycle arrest, for instance in MVM-infected cells, NS1 promotes the host cell cycle arrest in its S and G2 phases and upregulates the autophagy-inducing proteins, particularly TRIM23 (Lu-Jiao et al., 2025), and induces cytotoxicity in cells (Wang et al., 2025). It has also been found that HPV-B19 NS1 acts as a transcriptional transactivator by stimulating ATR, which in turn activates the ATR-CHK1-CDC25C-CDK1 pathway. In order to restrain the mitosis phase, activated ATR sends a signal to CDC25C, which as a result deactivates the cyclin B/CDK1 complex. This pathway arrests the cell cycle in its G2 phase and causes cell death (Xu et al., 2017). Similarly, the NS1 protein of CPV can induce a cell cycle halt in the S phase, facilitating viral multiplication (Op De Beeck et al., 2001), and it can also cause DNA damage to induce cell death at the G1/S phase of the cell cycle (Arora et al., 2021). Table 1 explains the different phases of the cell life cycle during which cell cycle arrest is provoked by the influence of parvoviruses NS1.

**Table 1:** Overview of cell cycle phase arrest associated with viral infection.

Virus	Cell cycle arrest phase	References
PPV	G1, G2	Chen et al. (2001)
CPV	G1, S, G2/M	Dai et al. (2020); Mattola et al. (2022)
MVM	S, G2	Adeyemi and Pintel (2014)
H-1PV	G2, M	Geiss et al. (2017)
HPV-B19	G1, G2	Xu et al. (2017); Morita et al. (2003)
H-Boca Virus	G2, M	Chen et al. (2010)
MEV	G1	Lin et al. (2019)

The functional variety of NS1 among parvoviruses affects their replication tactics, host-cell targeting, and therapeutic potential. Our comparative analysis Table 2 indicates that although NS1 in MVM and H1PV demonstrates significant oncolytic activity owing to elevated cytotoxicity in transformed cells, the NS1 variants of CPV and FPV exhibit wider tropism but diminished virulence, rendering them suitable for gene delivery. LuIII and PPV NS1 exhibit intermediate phenotypes with distinct immune-modulatory actions. These distinctions guide the selection of parvovirus backbones for particular translational objectives, such as oncolysis or vaccine production.

**Table 2:** Comparative functional investigation of NS1 among several parvoviruses (MVM, H1PV, CPV, LuIII, PPV, FPV), emphasizing differences in replication efficiency, host interactions, cytotoxicity, and therapeutic potential.

Parvovirus Species	NS1 Role in Therapy	Trial Phase	Indication	Key Outcomes	Safety Profile	Citations
H-1 Parvovirus (H-1PV)	NS1 is the major effector of viral cytotoxicity; it induces cell cycle arrest, apoptosis, and non-apoptotic death in tumor cells	Phase I/IIa (ParvOryx)	Recurrent glioblastoma multiforme	Demonstrated safety, tolerability, and first signs of anti-tumor activity	Well-tolerated, no significant adverse toxic effects	Bretscher and Marchini (2019)
Minute Virus of Mice (MVM)	NS1 is involved in cytotoxicity and DNA replication; preclinical focus; Apoptosis induction; and localization of viral genome.	In vitro	Rat fibroblast cells	Preclinical evidence of oncolytic activity	-	Xie et al. (2023); Mincberg et al. (2011); Majumder et al. (2020)
Lu-III	NS1 is involved in cytotoxicity; preclinical focus	-	-	Preclinical evidence of oncolytic activity	-	Afolami et al. (2018)
Canine Parvovirus (CPV)	Potential oncolytic agent by undergoing apoptosis and immune system modulation in neoplastic cells; Host translation shutoff.	Preclinical	Cancer treatment: glioblastoma, pancreatic cancer	Anti-tumor immune response alongside constructive targeting of malignant cells	Safety profile appears favorable, along with reduced off-target effects.	Arora et al. (2021); Wang et al. (2025)
Feline Panleukopenia Virus (FPV)	Currently, the NS1 protein of FPV is not utilized in direct therapies; however, it is critical for studying viral replication and immune system modulation.	Preclinical	Viral Pathogenesis: FPV Studies and Vaccine Development	Gaining insight into the mechanisms of viral replication and evasion of immune responses	Vaccinated populations maintain safety and efficacy, making them effective and safe.	Truyen et al. (2009)
Porcine Parvovirus (PPV)	Trigger apoptosis in PK-15 cells, it remains important for comprehending viral replication and immune system modulation.	Preclinical	Preventing reproductive failures and vaccine research	Understanding the mechanisms of viral replication as well as immune evasion techniques.	Safety and efficacy of PPV vaccines are excellent, with minimal adverse effects in vaccinated herds.	Streck and Truyen (2020); Zhang et al. (2019)

Significant distinctions highlight the context-dependent efficacy of these viruses in oncolytic and replication applications.

## 5 Apoptosis induction by NS1

**Figure 6:**
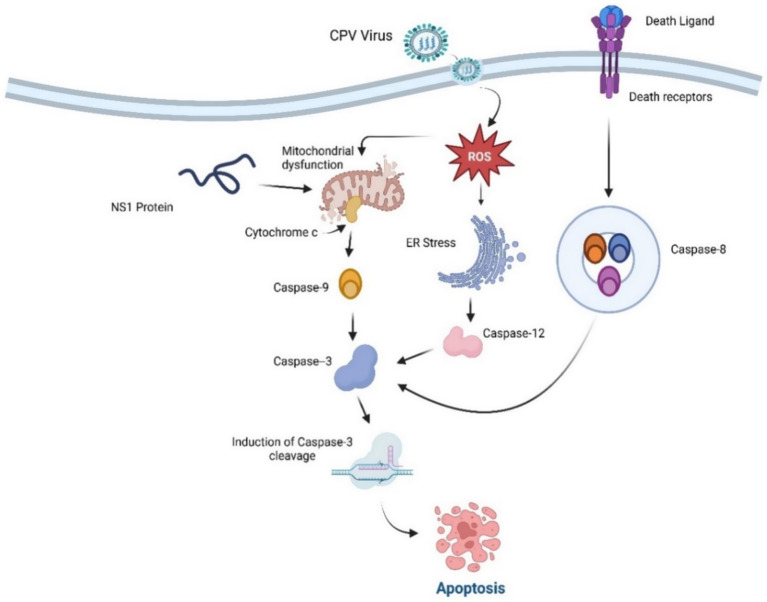
Apoptosis Induction by CPV NS1. NS1 induces ER and mitochondrial dysfunction and outer membrane permeabilization (MOMP), cytochrome c release, and the activation of the caspase cascade, culminating in apoptotic cell death. Both intrinsic (mitochondrial) and extrinsic (death receptor) mechanisms converge on caspase-3, resulting in apoptotic cell death.

Parvovirus NS1 plays a pivotal role in the induction of cell death following the cell cycle arrest. The mechanism of cell death induced by parvovirus varies based on the specific strain of parvovirus and the different cell lines infected (Afumba et al., 2022). Cell death is typically classified into three distinct types: apoptosis, oncosis, and necrosis. Among these, apoptosis is the most prevalent mechanism of cell death triggered by parvoviruses (Afumba et al., 2022) and is characterized as a controlled form of cell death following different pathways, while necrosis is characterized by its uncontrolled nature, signifying the terminal stage of cell death (D’arcy, 2019). During the entire process of apoptosis cell death, various cellular changes were also observed, including nuclear morphology such as nuclear and chromosomal DNA fragmentation and chromatin condensation (Zhang et al., 2019; Mészáros et al., 2017; Cao et al., 2020). Among the parvoviridae family species, the Non-structural 1 protein (NS1) is considered the most frequent apoptotic cell death-triggering agent of tumor cells. In Porcine parvovirus (PPV), NS1 can also trigger apoptosis in the host cell by following an intrinsic pathway involving mitochondrial-mediated cell death. This may also lead to tissue damage of the placenta, which eventually results in reproductive problems and collapse (Dai et al., 2020). It is thought that cell death in the premature stage of growth may be beneficial for combating the viral invasion and dissemination within the host cells, hence aiding in viral propagation (Kvansakul, 2017). NS1 of *human parvovirus* B19 has also been implicated in cell death by apoptosis cell death in erythroid lineage cells and triggering an autoimmune response (Jalali et al., 2022). Simultaneously, it has been reported that Hela cells infected by the *Canine parvovirus* undergo apoptosis induced by NS1 by interrupting the G1 phase. This process was influenced by the activation of caspase 9 and mitochondrial stress, leading to apoptotic cell death (Gupta et al., 2016; Doley et al., 2014). During CPV apoptotic cell death, host cells exhibited cell cycle arrest and DNA damage, with an elevation in the levels of the cyclin-dependent kinase (CDK) inhibitor p27 that was observed earlier than the cell cycle arrest (Arora et al., 2021). The activation of cyclins D/CDK4 and E/CDK2 was inhibited, which obstructed cells from transitioning out of the G1/S phase and entering the S phase. This obstruction was mainly attributed to the enhanced regulation of p27, a cyclin-dependent kinase inhibitor, which enforces cell cycle arrest. As a result, the prolonged cell cycle blockage activates apoptotic pathways leading to cell death (Léger et al., 2016). In CPV-infected cells, apoptotic cell death is often attributed to the buildup of reactive oxygen species (ROS) and the activation of caspase 3,8,9, and 12, along with endoplasmic reticulum pathways, as well as intrinsic and extrinsic pathways, which contribute to apoptosis induction (Arora et al., 2021). Figure 6 illustrates the mechanism involved in the apoptotic cell death induced by to CPV NS1 protein and several intracellular changes influenced due to it. Cells transfected with NS1 exhibited the hallmark feature of DNA fragmentation, indicative of endonuclease activation. Additionally, findings revealed that the p53 expression is generally sustained at very low levels of concentrations, but in response to certain conditions like DNA damage response DDR, hypoxia, and viral infection, p53 gene activation appeared, which caused the cell cycle regulation triggered via p21to seize or apoptotic cell death (Giglia-Mari et al., 2011). The activation of the p53 protein by *Canine parvovirus* in Madin Darby canine kidney (MDCK) cells has been revealed to play a role in the regulation of Bax and Bcl2 gene-associated factors, potentially through the mechanism of upregulation and downregulation, respectively which results in apoptosis (Zhao et al., 2016). Moreover, apoptotic characteristics include chromatin and nuclear condensation, cytochrome c release in the cytoplasm, and enhanced caspase-3 activation.

Considering the activation of caspase 3 to cleaved caspase 3 initiates the activation of the PARP protein, which facilitates the crucial role of the detection and repair of the disrupted segments of DNA strands. Along with it, the DNA damage response kinase (DDR) at the site of DNA damage leads to the suppression or reduction of cyclin-dependent kinase factors and results in cell cycle arrest and eradication of the damaged cells via apoptosis. Apoptosis is a regulated cell death that involves the activity of protease enzymes of the caspase family (Vitale et al., 2023). Several different types of caspases are activated in different deaths; for instance, caspase 8 is predominantly triggered in death receptor-mediated apoptosis, whereas caspase 9 is involved in intrinsic or mitochondrial-type death pathways and is observed to activate earlier in *Canine parvovirus* infection (Zhou et al., 2019). CPV prolongs the generation of viral lineage or progeny, leading to pathological outcomes characterized by cycle arrest and cell death due to DNA disruption and cell cycle interference caused by CPV (Morita et al., 2003; Nykky et al., 2010; Majumder et al., 2018). As well as CPV2 NS1 resulted in tumor cell breakdown, may have elicited an inflammatory response induced by a cross-presentation of tumor-associated antigens (TAAs) by dendritic cells (DC) and ensuing activation of tumor-specific CTLs (Moehler et al., 2005).

**Figure 7:**
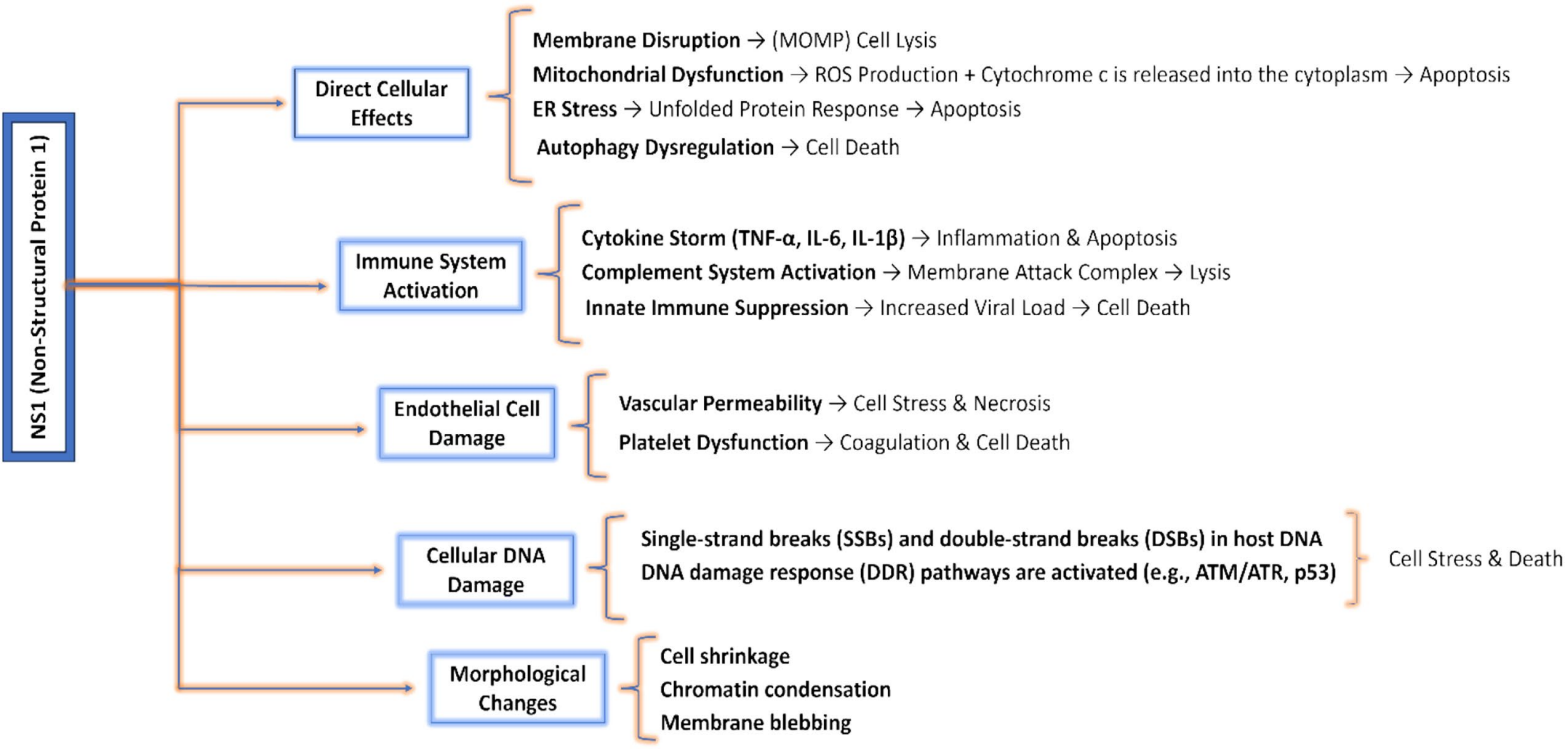
Pathways and mechanisms involved in cell death induced by NS1: highlights direct cellular effects including ER and mitochondrial effects; cellular DNA and endothelial damages like necrosis or cell stress and morphological changes like apoptosis, autophagy, and chromatin condensation.

In *Human Parvovirus* B19, Cell cycle arrest and apoptosis occur at an early stage, potentially resulting in a cytopathic effect. The cell cycle arrest takes place at the G2/M phase, primarily triggered by NS1 via the ATR-CHK1-CDC25C-CDK1 pathway as described earlier. It stimulates the C-terminal domain and facilitates the signaling pathway from ATR to CDC25C (Xu et al., 2017; Xu et al., 2019). During the infection caused by Human B19V, the phosphorylation of CDC25C inhibits the activation of CDK1, thereby resulting in the formation of an inactive B1-CDK1 complex (Xu et al., 2019). Upon being transferred into the nucleus, the inactive B1-CDK1 complex inhibits the progression of the cell from the G2 to M phase, resulting in G2/M cell cycle arrest and ultimately leading to apoptosis (Zou et al., 2018). NS1 possesses a fundamental characteristic of having a negligible cytotoxic effect on healthy cells, hence preventing the death of the surrounding cells and the deterioration or regression generated by NS1 is because of apoptosis, cytotoxic regression, and cell cycle arrest (Hristov et al., 2010; Nüesch Jr and Rommelaere, 2006). Several events and mechanisms are described in Figure 7 through which cell death is induced by the Non-structural protein 1 (NS1).

Table 3 highlights various parvoviral species and their NS1 proteins that have direct or indirect vital roles in oncolytic and viral cell death following different significant pathways, which have been studied and investigated in several research-based studies.

**Table 3:** Viruses and the role of their NS1 protein in cell death.

Virus Type	Host Species	NS1 Protein Function	Mechanism	Citations
Parvovirus H-1 (H-1PV)	Rats	Induces cytotoxicity and apoptosis in tumor cells. Cervical and pancreatic cancer cell death and lysis.	Activates intrinsic apoptosis pathways, DNA damage, immunosuppression, and oncotoxicity.	Bretscher and Marchini (2019); Moehler et al. (2005); Moehler et al. (2011); Angelova et al. (2014); Hamidi-Sofiani et al. (2022); Li et al. (2013)
Canine parvovirus (CPV)	Canine	Induce programmed cell death oncolysis.	Can induce DNA damage and apoptotic cell death pathways.	Nykky et al. (2010); Pan et al. (2012); Arora et al. (2024); Arora (2021)
porcine parvovirus (PPV)	Swine	Triggers apoptosis in PK-15 cells and placental tissue damage and cytotoxicity.	Mitochondrial dysfunction intrinsic pathway, caspase activation, and upregulation of tumor necrosing factor.	Zhang et al. (2019); Jin et al. (2020); Zhao et al. (2016); Ma et al. (2020); Puttaraksa et al. (2019)
Bovine parvovirus (BPV)	Bovine	Induces cell death and cytotoxicity. Necrosis in bovine tracheal cells	Follows the apoptosis and necrosis signaling pathway and induces caspase activation.	Nykky et al. (2010); Abdel-Latif et al. (2006); Deng et al. (2017)
Adeno-associated viruses (AAV)	Humans and some other primate species	Induces cell death in liver cancer cells and human 293 cell line	PDK1 signaling pathway; apoptosis induced by cadmium and non-structural protein combination	Witzigmann et al. (2021); Zhou and Trempe (1999)
Human B19 Parvovirus	Human	Causes cytotoxicity for cell death in tumor cells	Induce DNA damage and apoptosis by caspase activation.	Jalali et al. (2022); Moffatt et al. (1998); Poole et al. (2011); Kivovich (2011)
Aleutian Mink disease virus (AMDV)	American mink	Induces apoptosis in tumor cells	Triggers mitochondrial stress and apoptotic cell death pathway	Chen and Qiu (2010)
Minute Virus of Mice (MVMp)	Laboratory mice	Induces apoptosis in A9 fibroblasts	ATM signaling, Interference with CKIIα signaling	Nüesch Jr and Rommelaere (2006); Kailasan et al. (2015)
Rat parvovirus (RPV)	Laboratory rats	Suppressed tumorigenicity	Induce tumor suppressor gene p21 and induce apoptosis.	Iseki et al. (2005); Abdel-Latif et al. (2006)
Feline panleukopenia virus (FPV)	Feline	Feline Lymphoid cells	Apoptosis induction	Ikeda et al. (1998)
Boca Virus (HBoV)	Humans specifically children	Susceptible apoptosis inducer and autophagy	Mitochondrial-mediated apoptosis and DNA damage	Chen et al. (2010); Luo et al. (2011); Sun et al. (2013); Zhu et al. (2019)
Luiii Parvoviruses	Rodents	Induce cell death	Not defined yet	Kailasan et al. (2015)
Goose Parvovirus (GPV)	Gooselings and ducklings	Induces apoptosis in Goose embryo fibroblasts	Apoptosis-inducing factors (AIFs)	Yan et al. (2021)
Mink Enteritis Virus (MEV)	Minks	Induced apoptosis in human embryonic kidney (HEK293T) and F18 cells,	Mitochondrial Pathway, Bax/Bcl2, MAPK and p53.	Lin et al. (2019)

## 6 Immunomodulation induced by NS1 in conjunction with adjuvants poly (I: C)

Oncolytic immunotherapy focuses on the application of oncolytic viruses (OVs), including *Canine parvovirus*, Newcastle disease virus (NDV), and others (Coleto et al., 2024). In boosting the immune response, cellular proteins (Liao et al., 2019) play a significant role, like NS1 of influenza A virus, which inhibits the function of RIG and RIG-dependent activation of NF-κB (Maarouf et al., 2018) and can also trigger the formation of autophagosomes (He et al., 2023). In parvoviruses, the NS1 protein is also of significant importance for tumor therapeutic capabilities. Along with the eradication of tumor cells through various mechanisms, NS1 has also demonstrated the capability to stimulate the immune system (Gupta et al., 2016; Bär et al., 2013; Nüesch Jr and Rommelaere, 2006; Gupta et al., 2015; Di Piazza et al., 2007). Both of these situations reveal the presentation of tumor-associated antigens (TAAs) to antigen-presenting cells, resulting in the establishment of a cytotoxic T-cell response (CTL response; Chen et al., 2001; Guermonprez et al., 2002; Festjens et al., 2006). However, the activation of immune cells by the NS1 protein is inherently weak (Santra et al., 2014), but the incorporation of an adjuvant may enhance its efficacy. Furthermore, numerous cancers exhibit limited immunogenicity, necessitating the incorporation of an adjuvant to elicit robust anti-tumor T-cell immune responses. Significantly, NS1 enhances anti-tumor immunity: research in animal models reveals that NS1 administration augments the infiltration of immune cells, including CD4 + and CD8 + T cells and natural killer (NK) cells, into tumor tissue, and elevates Th1 cytokines such as IFN-γ and IL-2, signifying a vigorous cell-mediated immune response (Gupta et al., 2016).

The H-1 parvovirus (H-1PV) demonstrates oncosuppressive effects involving two aspects: oncotoxicity and immunostimulation. A preliminary clinical investigation of H-1PV treatment in glioma patients has demonstrated intratumoral production of the viral oncotoxic protein NS1 and infiltration of immune cells. The experimental study revealed enhanced infiltration of CD8^+^ T cells and macrophages in tumor tissue subsequent to H-1PV therapy, accompanied by activation of interferon-stimulated genes and proinflammatory cytokines such as IL-6 and TNF-α (Geletneky et al., 2017). Multiple *in vitro* studies have consistently demonstrated that H-1PV infection of human peripheral blood mononuclear cells (PBMCs) is non-productive; however, initial stages of viral replication transpire. Further analysis has identified B-cells, macrophages, and natural killer (NK) cells as the primary immune cell subpopulations infected by H-1PV, with infection in activated cells leading to the induction of tumor necrosis factor (TNF)-alpha, interferons (IFN), and interleukins (IL) 2, 4, and 10 (Geletneky et al., 2015).

In CPV 2, NS1 and poly (I: C) individually can cause cell death in 4 T1 tumor cells induced in mice, but if they are administered in combination, they can robustly activate the immune system. In cancer therapeutics, vaccine adjuvants targeting toll-like receptors (TLRs) exhibit significant potential because of their ability to identify pathogen-associated molecular patterns (PAMPs) and boost innate and adaptive immunity (Kaur et al., 2022). It is well-known that the Toll-like receptor 3 (TLR3) ligand polyinosinic-polycytidylic acid [poly (I: C)] can stimulate the production of inflammatory cytokines and type I interferon (IFN). Poly (I: C) can also augment the production of type 1 interferons via its interaction with the cytoplasmic receptor, melanoma differentiation-associated protein 5 (MDA-5). The TLR3 and MDA-5 pathways, upon stimulation with poly (I: C), can induce apoptosis, produce cytokines and chemokines, and facilitate the maturation of dendritic cells (Cheng and Xu, 2010). In addition to its function in eliciting an immune response, recent studies have revealed that poly (I: C) has also been demonstrated to directly induce apoptosis in a wide variety of tumor types both in vitro and *in vivo* (McBride et al., 2006; Banz et al., 2012). This renders poly (I: C) a compelling potential treatment choice for tumors. CPV2 also triggers a potent anti-tumor immune response by generating antibodies against tumor-associated (TA) genes (Arora et al., 2021). Upon induction of apoptosis or cell lysis by CPV2 NS1, the generated tumor antibodies and TA genes are presented to MHC-I by antigen-presenting cells (APCs). It stimulates tumor-specific cytotoxic T lymphocytes (CD8+) and natural killer cells, which are responsible for tumor elimination. T helper cell activation assists in the CTL proliferation and presenting antigens to B cells, which results in the development of antitumor antibodies and stimulates the immune response (Arora et al., 2021). Similarly, in *human parvovirus* B19, NS1 stimulates the host immune response by activating the type I interferons (Wu et al., 2016). These activated interferons play a vital role in the immune stimulation of the cells.

## 7 Oncolytic role and mechanism of parvoviral strains and NS1

### 7.1 H-1 parvovirus (H-1PV) NS1

Parvovirus H-1PV has been recognized for targeting and destroying a variety of human tumor cells encompassing melanoma, hepatoma, gastric, colorectal, cervical, and pancreatic cancers (Moehler et al., 2011). H-1PV is being explored as an anticancer agent due to its non-pathogenic nature for humans and its oncotropic and oncosuppressive attributes. The NS1 of the H-1PV is considered to be the key mediator of the cytotoxic effects on cancer cells (Hristov et al., 2010). H-1PV selectively infects and eradicates cancer cells while preserving normal cells, rendering it a promising candidate for oncological treatment. The H-1 Parvovirus causes tumor cell death by a multimodal mechanism that includes direct cytotoxicity, immune response regulation, and tumor microenvironment (TME) alteration (Iseki et al., 2005). The NS1 protein is integral to this process, inducing cytotoxic effects on tumor cells via many methods. A key process involves the activation of apoptosis and the activation of caspases 3 and 9 in neoplastic cells. Studies demonstrate that H-1PV NS1 induces cell death in human promonocytic 937 cells by following significant signaling pathways, including the activation of PARP to cleaved PARP and stimulation of tumor necrosis factor-alpha (TNF-α) therapy. This mechanism also involves the activation of CPP32 ICE-like cysteine proteases, which cleave the enzyme poly (ADP-ribose) polymerase and ultimately result in distinct apoptotic morphological alterations (Ba et al., 1998). The oncolytic attributes of H-1PV were also verified in in vivo trials in nude mice models against MKN28, SGC7901, and MKN45 human gastric cancer cell lines and transfected with the pcDNA3.1 plasmid of H-1PV NS1. The results demonstrated the NS1-associated suppression of the tumor with the reduction in the proportion of CD44-positive cells (Wang et al., 2012). Furthermore, H-1PV NS1 also plays a role in modulating the activation of the immunological response. Following infection, H-1PV augments the phagocytosis, maturation, and cross-presentation functions of dendritic cells (DCs), thereby leading to significantly improving anti-tumor immunity (Bretscher and Marchini, 2019; Moehler et al., 2005). It also selectively activates helper and non-regulatory CD4 + T (Bhatt and Daemen, 2024) by the enhancement of activation marker expression (CD 69 and CD 30) alongside the secretion of both effective Th 1 and Th2 cytokines (IL-2, IFN-γ and IL-4) thus exhibiting its anticancer attributes in xenotransplanted human nasopharyngeal carcinoma, in a SCID mouse model (Moralès et al., 2012).

### 7.2 Minute virus of mice (MVM) NS1

The *Minute Virus of Mice* (MVM) has gained interest for its potential application as an oncolytic immunotherapeutic agent (Angelova et al., 2023), owing to the tumor cell apoptosis which is triggered through the operation of its nonstructural protein. Studies revealed that the MVM infection transformed into the cells leads to the elevation of the cytolytic NS1 protein that triggers the rat fibroblast cell death process influenced by cellular factors like apoptosis due to the activation of caspase 3 and 9, but not caspase 8 (Mincberg et al., 2011). It also demonstrated that MVM NS1 can induce cell death in tumor cells lacking p53, by following the ATM/ATR kinase pathway induced due to DNA damage response. MVM SAT protein, which is ideally a late Non-structural protein, induces lysis of the cells by the formation of viroporin (viral-encoded transmembrane protein) like structures and forms pores in the plasma membrane to make it permeable. Knockout of the SAT in the genome can decrease the cell lysis capability of the MVM virus. MVM selectively infects and eradicates malignant cells, utilizing specific characteristics of these cells for its multiplication and lethal effects. It has been shown that NS1 can elicit an irreversible stress response in the endoplasmic reticulum, resulting in apoptosis in pathogenic cells (Bretscher and The, 2021). Depending on the cell’s transformation phase, the autonomous parvovirus minute virus of mice’s nonstructural protein NS1 can interrupt cell division and even induce cell death. The expression of NS1 is associated with the suppression of DNA replication and disruption of the host cell cycle, resulting in cell arrest during the S phase. This disturbance frequently precedes cellular apoptosis (Op De Beeck and Caillet-Fauquet, 1997). Regarding cellular structural changes, MVM NS1 can cause alterations in the chromatin architecture of the host cell, leading to substantial DNA damage. From past studies, there was an evident role played by the minute virus of mice (MVM) nonstructural (NS) proteins in viral DNA replication, promoter regulation, and cytotoxicity, especially in transformed cells. Along with it, It appears that NS2 did not work in tandem with NS1 because cytotoxicity was achieved solely by NS1. The correlation between cell death and oncogene expression suggests that NS1 is only toxic when oncogene-induced cellular factors are present (Mousset et al., 1994).

### 7.3 Porcine parvovirus (PPV) NS1

The nonstructural protein 1 (NS1) of Porcine Parvovirus (PPV) has been investigated for its function in promoting cell death, especially concerning malignant cells. The mechanism by which PPV NS1 induces cell death is quite complex, but some studies indicate that the expression of PPV NS1 promotes apoptosis in host cells, including tumor cells. It includes the activation of the intrinsic apoptosis pathway, which is mediated through mitochondrial signaling and caspase activation (Zhang et al., 2019). Research findings demonstrate that the expression levels of IL-6 and tumor necrosis factor-alpha can be upregulated by the PPV NS1 protein in a dose-dependent manner. Additionally, it was observed that the PPV NS1 protein induced the phosphorylation of IκBα, which in turn resulted in the phosphorylation and nuclear translocation of NF-κB. Ultimately, these processes boosted the immune response, Apoptotic processes, and the induction of pro-inflammatory cytokines, which were influenced by the activation of these pathways (Jin et al., 2020). This mechanism can also activate the TLR-2 receptors, which leads to the induction of the production of cytokines.

### 7.4 Human B19 parvovirus (HPV-B19) NS1

The oncolytic attributes of the HB19 parvovirus NS1, followed by different mechanisms, have been revealed in several mammalian cell lines, like erythroid lineage cells (Yaegashi et al., 1999) and human liver cancer carcinoma (HepG2), by following the intrinsic apoptosis mechanism and cellular DNA damage (Kivovich et al., 2011). To further investigate and validate the mechanism of cellular toxicity and apoptosis cell death, the NS1 gene in a cytomegalovirus episomal vector was transfected into monkey epithelial COS-7 cells, and found that the p53 and cell cycle kinase inhibitors, including p16 and p21, were upregulated in the NS1-transfected cells. It also revealed that NS1 also induced the activation of caspase 3 and 9 along with the upregulation of pro-apoptotic members Bcl-2, Bax, and Bad, which clarifies that HPV-B19 NS1 induced cell death by following the mitochondrial apoptotic pathway (Hsu et al., 2004). An augmented production and expression of IL-6 was also noticed in the B19 NS1-transfected COS-7 epithelial cells which may play a role in the pathogenesis of autoimmune disorders (Hsu et al., 2006).

## 8 Harnessing parvoviruses and NS1: combination and advanced gene therapy

Parvovirus NS1 protein has been widely recognized in the research domain because of its multifunctional attributes, specifically oncolytic characteristics; however, its efficacy can be limited due to the influence of factors, including tumor resistance mechanisms or microenvironment. Therefore, combinational therapy or genetically engineered NS1 of several parvoviral species, specifically H-1PV combined with adjuvants like gemcitabine (Angelova et al., 2009), Bevacizumab (Geletneky et al., 2015), and Nivolumab (checkpoint blocker; Angelova et al., 2021) has been considered as an optimized, progressive, and novel therapeutic source against tumor cells. A combination of ionizing radiation and H-1PV revealed enhanced cytotoxicity in various glioma-derived cell lines, including radio-resistant cells with an increase in the elevated NS1 compared to the individual agent-treated cells (Marchini et al., 2015). Pancreatic and cervical carcinoma cells were also treated with a therapeutic combination of H-1PV and histone deacetylase inhibitors (HDACIs) such as valproic acid (VPA) and found that acetylation of NS1 lysine residues at K85 and K257 can upregulated NS1 mediated transcription and cytotoxicity augmented with the aid of VPA therapy (Li et al., 2013; Luo et al., 2022). These findings imply an assessment of H-1PV/VPA clinical trials against cancer cells. Another combinational therapy involves H-1PV and interferon gamma-mediated treatment. IFNγ suppresses peritoneal ductal adenocarcinoma (PDAC) in the rat orthotopic pancreatic cancer model. Functionally, the H-1PV and IFNγ combination augments the macrophage and splenocyte production against pancreatic cancer cells (Grekova et al., 2011). To augment its immunogenicity, CpG motifs were incorporated into its genome, and as a result, the engineered variants JabCG1 and JabCG2 elicited enhanced TLR9-mediated immunological responses. In a rat lung metastasis model, JabGC2 enhanced immunogenicity by promoting dendritic cell activation and decreased metastatic rates, thereby illustrating the immense potential of CpG-enriched oncolytic parvoviruses’ cancer immunotherapeutic capabilities (Bhatt and Daemen, 2024; Raykov et al., 2008). As previously stated, poly (I: C) was also proved to be a highly effective adjuvant for *Canine parvovirus* NS1 to induce immunogenic cell death in 4 T1 cells. For clinical application, the genetically engineered NS1 gene (VIVO.ns1) has revealed a tremendous anti-tumor response against canine transmissible venereal tumor (CTVT). The NS1 gene was also used with the combination of chicken anemia VP3 gene (VIVO.vp3), but the oncolytic outcomes of the NS1 gene solely treated group were better compared to the NS1 gene + VP3 gene combination group (Bhat et al., 2017). Past studies also revealed parvoviral NS proteins as genetic modulation of tumor suppressive pathways through targeted epigenetic regulation. RPV NS protein upregulated the ciliary neurotrophic factor receptor alpha (CNTFRα) and enhanced its histone acetylation in the host cell genes, and suggested an epigenetic mechanism of oncosupression in rat thymic lymphoma C58 (NT) cells. This genetic modification can revert the tumor malignancy to benignancy (Iseki et al., 2005). Furthermore, to validate the therapeutic combination of MVM NS1 cytotoxic and oncolytic effects experimentally, a controlled hormone inducible promoter (dexamethasone) was employed to upregulate the non-structural proteins in the MVM-infected human and rat fibroblasts transfected with NS gene, and as a result, dexamethasone promoted the synthesis of NS protein which caused the cell death rapidly (Anouja et al., 1997). Moreover, in Human B19 parvovrius, mutations in the NS1’s nucleoside triphosphatee binding domain prevented the apoptosis without impacting NF-kB mediated IL6 activation, indicating a functional uncoupling of transcriptional activation (Moffatt et al., 1998).

**Figure 8:**
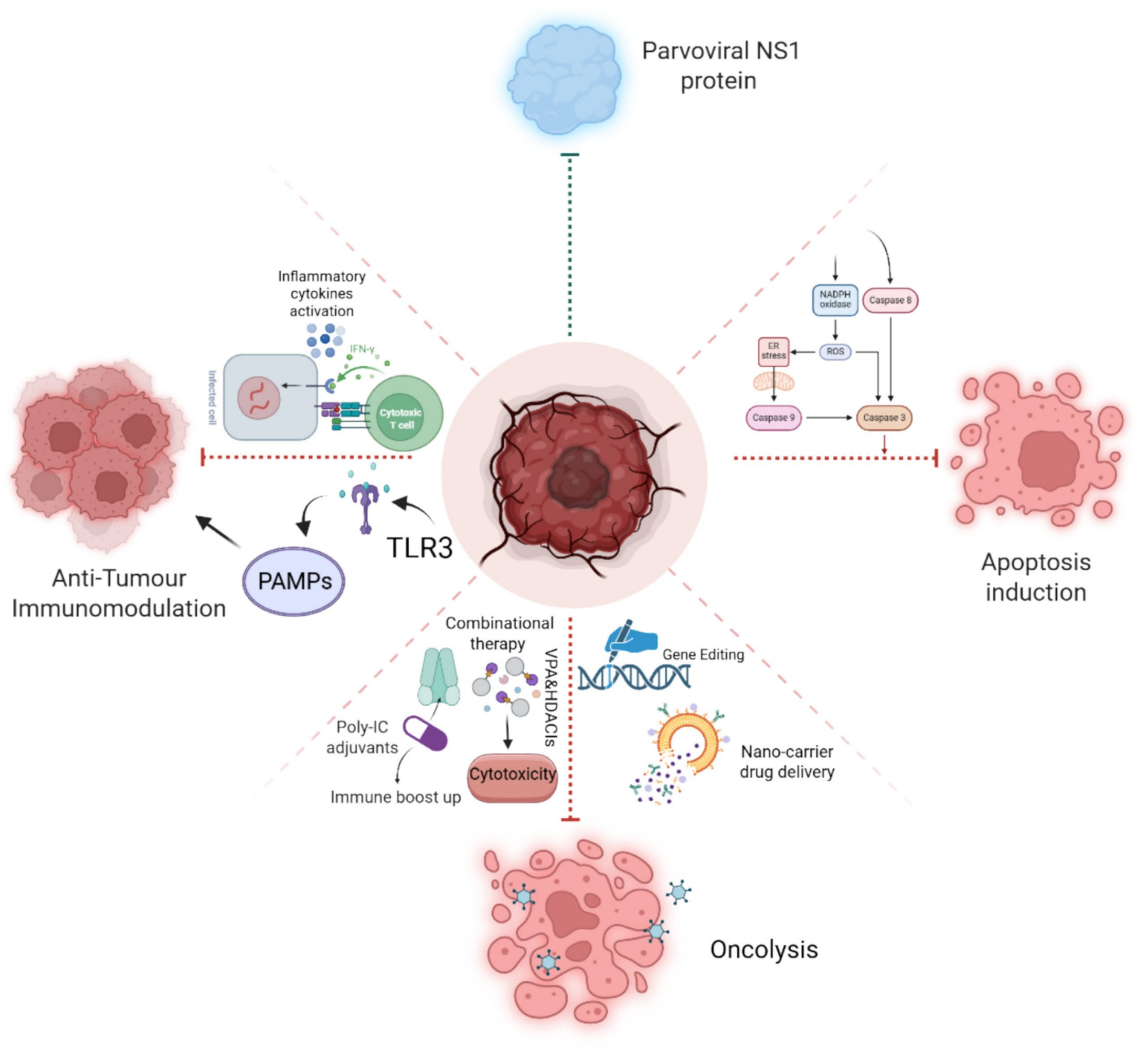
Schematic illustration of NS1-induced pathways, Apoptosis induction by caspase cleavage, Anti-tumor immunomodulation by the activation of inflammatory cytokines and PAMPs, and NS1’s role in oncolysis with the aid of genetic engineering techniques and combinational therapeutics strategies.

Since parvoviruses can act as significant gene transfer vectors, they have also emerged as a promising agent to target tumor cells. Specifically, AAV has gained significant attention as a potential gene transfer vector, enhanced comprehension of vector genome metabolism, and the transduction mechanism of AAV vectors can facilitate progress in the creation of more advanced and resilient AAV vectors that may have more transduction efficiency and more specificity in targeting tumor cells (Gupta, 2022). Later investigations demonstrated that capsid-optimized AAV vectors expressing ovalbumin (OVA) and tumor-associated genes were used to validate immunotherapy of the cancer cells by the activation of protective T cells in peripheral blood in mouse models. The findings revealed that these OVA-specific T cells had a high compatibility to kill mouse prostate cancer cells RM1 (Pandya et al., 2015). AAV vectors have also been extensively employed in several tumor therapies, including peripheral nerve tumors (Greven et al., 2023), airway epithelial cell transduction (Halbert et al., 2001), and hepatocellular carcinoma (Meumann et al., 2022). Conclusively, Parvoviruses, especially via the function of NS1, demonstrate considerable potential as tumor-selective agents in cancer treatment. Their inherent oncotropism and cytotoxicity, along with progress in genetic engineering, facilitate the creation of customized vectors that exhibit improved specificity and safety. Integrating parvoviral therapy with immunotherapy, chemotherapy, or radiotherapy presents a synergistic strategy to address tumor resistance. Future efforts must prioritize the optimization of delivery systems, clarification of NS1 mechanisms, and enhancement of combinational strategies to effectively translate these tools into precision oncology platforms (Figure 8).

## 9 Discussion

The NS1 protein of Parvoviruses has emerged as a potential target for cancer therapy because of its capacity to specifically induce apoptosis in tumor cells while preserving healthy tissues. This study consolidates existing knowledge regarding the molecular pathways involved in NS1-mediated apoptosis and its prospective application as an oncolytic drug. NS1, a nuclear phosphoprotein including three essential domains—N-terminal origin-binding, helicase, and C-terminal transactivation domains—functions in both viral replication and host cell manipulation (Xie et al., 2023; Niskanen et al., 2010). NS1 arrests the cell cycle at the G1/S phase in CPV, facilitating viral DNA replication while activating intrinsic apoptotic mechanisms via mitochondrial depolarization, buildup of reactive oxygen species (ROS), and the activation of caspases, especially caspase-9 (Gupta et al., 2016; Saxena et al., 2013). Comparisons of NS1 proteins among various parvoviruses, including Rat parvovirus (H1-PV) and *Human parvovirus* (B19V), demonstrate common apoptotic effects alongside species-specific differences in cell cycle arrest phases. CPV-NS1 predominantly promotes G1/S arrest, whereas B19V-NS1 inhibits the G2/M phase through the ATR-CHK1-CDC25C pathway (Xu et al., 2017). These disparities underscore the necessity for customized therapy approaches. Structural studies highlight the significance of NS1’s helicase domain in unwinding viral DNA and its transactivation domain in regulating host gene expression, hence augmenting viral replication and cytotoxicity (Tewary et al., 2015). Notwithstanding these advancements, difficulties persist. Contemporary research predominantly relies on *in vitro* and small-animal models, resulting in deficiencies in comprehending NS1’s pharmacokinetics, biodistribution, and long-term safety in humans. Subsequent research should emphasize *in vivo* evaluations of NS1 delivery mechanisms, including viral vectors or nanoparticles, to enhance tumor targeting and reduce off-target effects (Miao et al., 2022; Feng et al., 2014). In addition to direct lethal effects, NS1 induces immunogenic cell death (ICD), which releases tumor-associated antigens and danger signals that may synergistically boost anticancer immunity when used with immunotherapies such as checkpoint inhibitors. The reported impairment of homologous recombination indicates a potential for combination with PARP inhibitors in malignancies lacking in DNA repair. Furthermore, NS1’s pro-inflammatory properties may alter the immunosuppressive tumor microenvironment, mitigating immune evasion strategies employed by regulatory T cells or myeloid-derived suppressor cells.

Furthermore, investigating NS1 in combination with checkpoint inhibitor drugs, chemotherapy, or radiation may leverage synergistic processes to surmount tumor resistance (Bretscher and Marchini, 2019; Hristov et al., 2010). Parvovirus-derived vectors hold significant potential for gene therapy in oncological conditions and genetic abnormalities in humans. Parvoviruses were effectively utilized in experimental treatments on animal models of human glioma, neuroblastomas, lymphomas, pancreatic carcinoma, carcinomas, and breast cancers. ParvOryx is the inaugural oncolytic formulation derived from H-1PV; its phase I/IIa clinical studies in glioblastoma multiforme patients are now underway (Loktev et al., 2012). Engineered NS1 variations or targeted delivery systems could enhance tumor selectivity, while strategic combinations with immunotherapy, radiation, or small-molecule inhibitors may address the limits of monotherapy. These combinatorial strategies utilize complementary mechanisms to enhance efficacy and minimize negative effects.

Viral gene therapy also holds great potential for cancer treatment due to its ability to specifically target tumor cells while minimizing off-target effects. Traditional oncolytic viruses face safety concerns, prompting a shift towards using viral genes such as the NS1 protein from *Canine parvovirus* (CPV) for cancer therapy. The characteristics discovered in several experimental investigations indicated that the *Canine parvovirus* NS1 gene possesses apoptotic action in the regression of mammary gland tumors (Verma et al., 2014). The use of the NS1 gene in gene-based therapy offers several advantages, including targeted cancer cell destruction without the safety risks of viral replication. Significantly, recent research indicates that NS1 may function synergistically with various therapeutic approaches, such as radiation or small-molecule inhibitors that intensify DNA damage or alter the tumor microenvironment. However, challenges persist, particularly in the practical administration of NS1 and in comprehending the range of tumor types most vulnerable to its effects. The field would benefit from advanced vector engineering techniques to augment NS1 expression in vivo, with comprehensive transcriptome and proteomic investigations to elucidate resistance mechanisms and refine patient selection. Overall, Parvoviral NS1 is a promising yet underutilized oncolytic agent, necessitating comprehensive preclinical and translational research to fully exploit its anticancer capabilities.

### 9.1 Translational challenges and strategies to overcome

Despite its promising preclinical performance, the transition of NS1-based oncolytic virotherapy to clinical use encounters numerous substantial obstacles. The effective delivery of viral vectors to tumor sites is a significant challenge, as systemic injection frequently leads to fast clearance, off-target effects, and restricted tumor penetration, especially in varied tumor microenvironments. Tumor heterogeneity presents a challenge, as the differential expression of viral entry receptors and the heterogeneous cellular composition within tumors might result in variable viral infection and therapeutic outcomes. Additionally, the immunogenicity of NS1 and the viral vector might provoke strong antiviral immune responses that may prematurely destroy the virus, hence diminishing therapeutic efficacy. Furthermore, cancers may acquire resistance mechanisms via antiviral signaling pathways or immune evasion tactics that hinder viral multiplication and oncolysis. To tackle these challenges, various strategies are being explored, including the implementation of targeted delivery systems like nanoparticle carriers or cell-based vectors to improve tumor-specific viral delivery, the engineering of viral vectors to circumvent immune detection or to express immunomodulatory molecules that alter the tumor microenvironment, and the combination of virotherapy with immune checkpoint inhibitors or other immunotherapies to enhance antitumor immune responses. Ultimately, a comprehensive strategy that incorporates enhanced vector design, optimized delivery, and combinatorial therapies will be crucial to surmount these translational obstacles and fully realize the clinical potential of NS1-based oncolytic virotherapy.

## 10 Future perspectives

Exploring NS1’s capacity to alter the tumor microenvironment, particularly its influence on immunosuppressive cells and cytokine signaling, could provide valuable insights for enhancing its oncolytic efficacy. Comprehensive in vivo studies must be focused on assessing NS1’s effectiveness in animal models as well as evaluating its bioavailability, tumor targeting, and safety profile. Additionally, integration of NS1 with other therapies like immunotherapy and chemotherapy may augment its anti-tumor efficacy, while personalized medicine strategies could further refine its therapeutic potential. Regarding clinical outcomes, it could be enhanced by integrating NS1-based oncolytic virotherapy with combinational therapeutic agents such as immune checkpoint inhibitors or chemotherapy. Ultimately, the therapeutic application of NS1-based oncolytic approaches relies on the development of genetic modifications or delivery systems to enhance NS1 stability, tumor targeting, and systemic administration.

**Figure 9:**
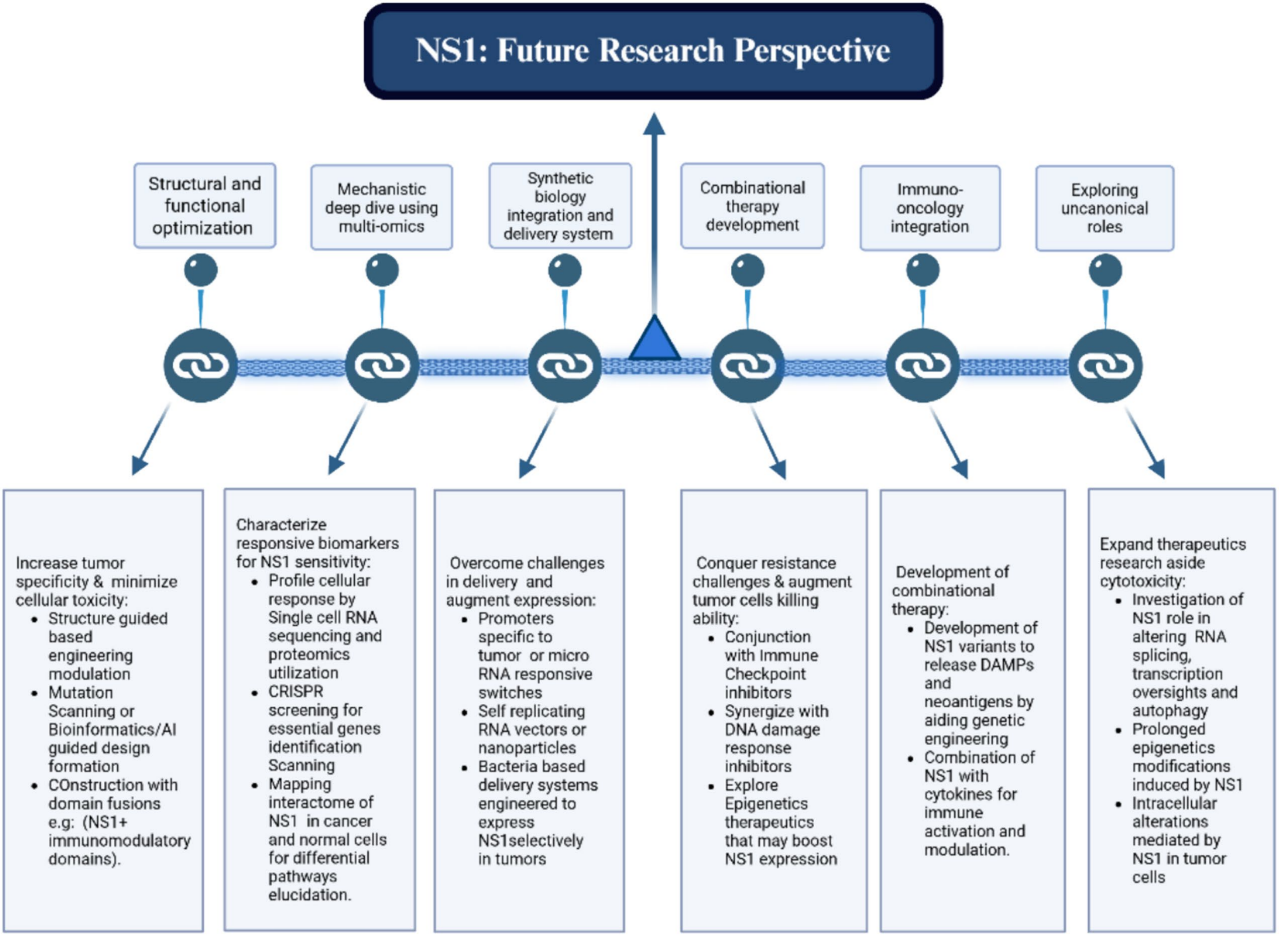
Summary of the proposed framework, highlighting potential future possibilities such as scalability enhancements, cross-domain applications, and integration with advanced technologies.

The details elaborated in Figure 9 establish a foundation for numerous promising future avenues. This encompasses broadening the methodology to more areas, augmenting scalability and resilience, and incorporating emerging technology for improved performance. By delineating these viewpoints, we seek to promote ongoing inquiry and advancement in this domain.

## 11 Conclusion

In summary, the shift to viral genes, like NS1, in cancer therapy presents a safer and more targeted alternative to traditional oncolytic virus treatments. CPV and other parvoviruses NS1 signifies a transformative advancement in oncolytic therapy, providing a focused and multi-faceted strategy for cancer treatment. Its capacity to elicit caspase-dependent apoptosis, enhance anti-tumor immunity, and synergize with adjuvants establishes it as a multifaceted instrument in cancer. The parvoviral NS1 protein emerges as an exceptionally versatile oncolytic drug, integrating direct tumor destruction with substantial immune activation. This dual functionality establishes it as a transformative element in cancer therapeutics. Its capacity to selectively induce DNA damage and immunogenic cell death in malignant cells, while preserving healthy tissues, presents a promising therapeutic opportunity for addressing aggressive or treatment-resistant malignancies.

*In vivo* studies and combination therapies are the key to unlocking their full potential in future research and clinical applications. Transitioning NS1 from laboratory to clinical application necessitates thorough preclinical validation and inventive approaches to overcome delivery and safety issues. Leveraging advancements in virology, immunology, and nanotechnology, NS1-based therapeutics have the potential to transform cancer treatment, addressing the demand for safer and more effective alternatives to traditional oncolytic viruses. The increasing interest in virotherapy and precision oncology necessitates additional investigation into NS1’s molecular mechanisms, therapeutic delivery, and clinical applications, which could facilitate the creation of innovative, tumor-selective anticancer strategies.
